# Supplementary figures and images for: Secreted ISG15 induced by Chlamydia trachomatis infection exerts immunomodulatory effects on IFN-γ defense and inflammation
Source: PLoS Pathog. 2025 Jul 8;21(7):e1013315. doi: 10.1371/journal.ppat.1013315 (PMC12373280; doi:10.1371/journal.ppat.1013315)

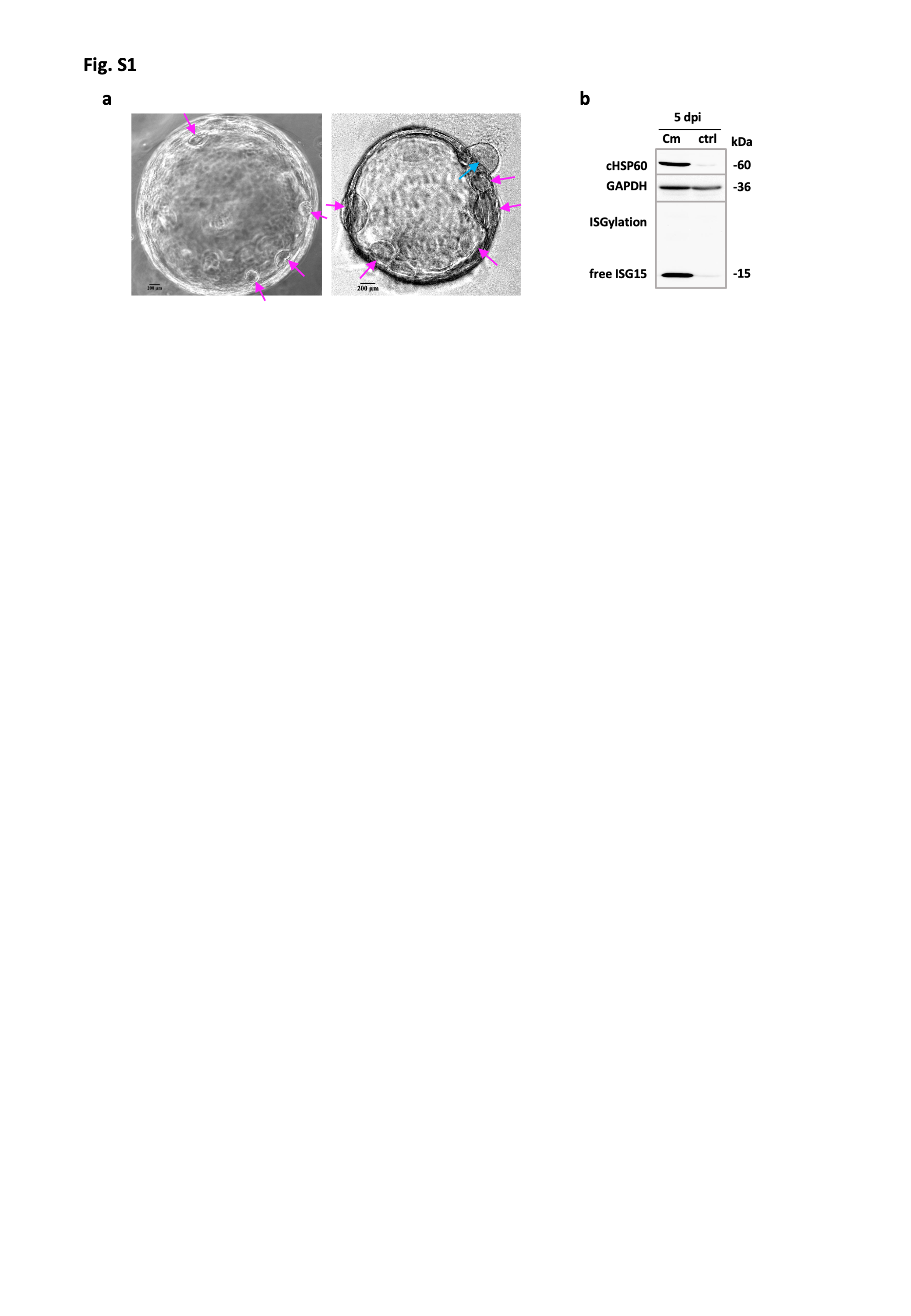

Supplement: S1 Fig — The release pattern of Ct inclusions in the mFGT organoids was observed as apical release into the organoid lumen (pink arrows) as well as basolateral release into the extracellular matrix (blue arrows), primarily through apical release. b. ISG15 expression in mFGT organoids infected with Cm for 5 days. ISG15, HSP60, and GAPDH expression of infected and non-infected mFGT organoids were analyzed by Western blotting. A representative blot from three independent experiments is shown. (TIFF) [file ppat.1013315.s001.tiff]

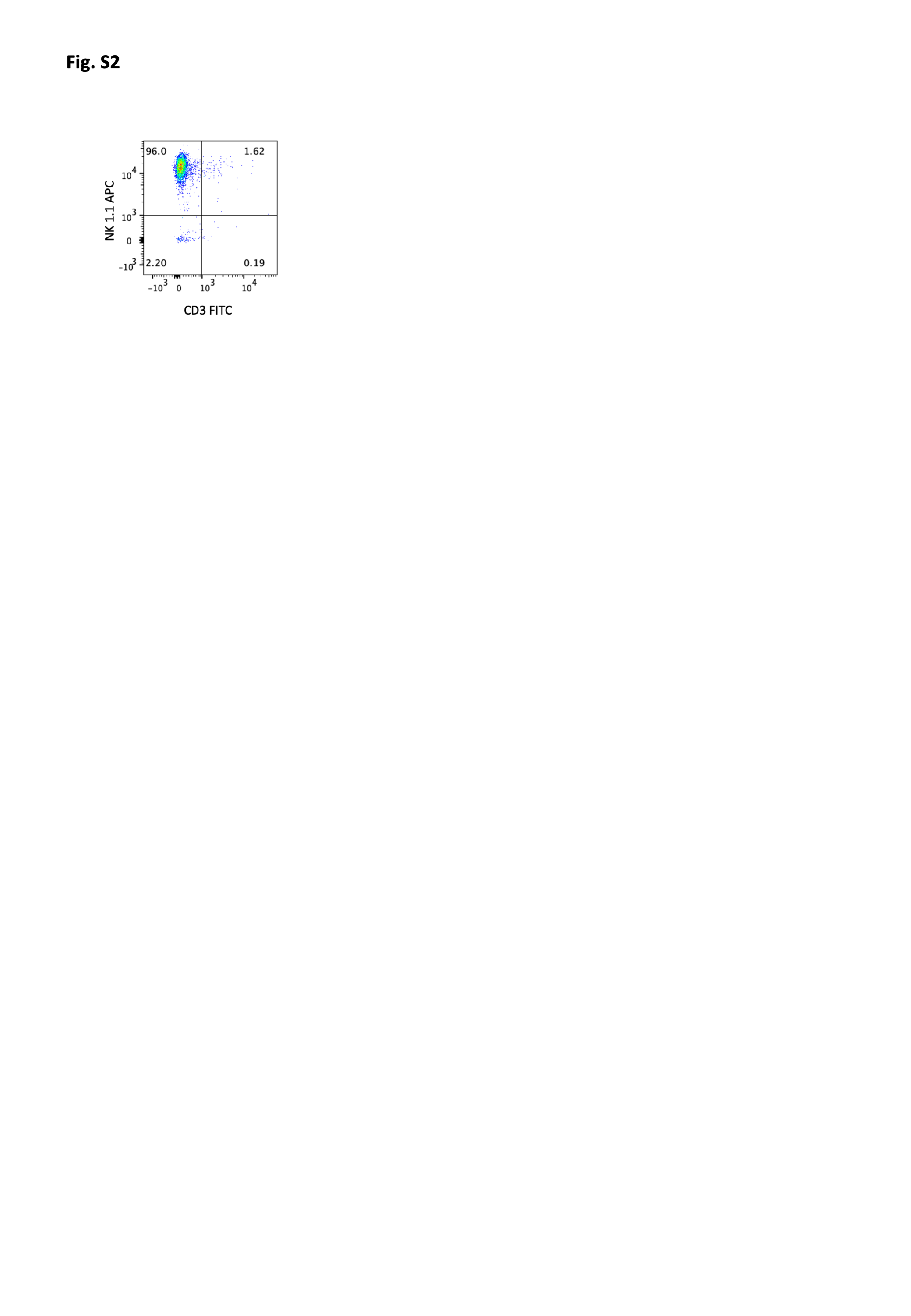

Supplement: S2 Fig — Primary NK cells were enriched from mouse splenocytes using negative magnetic selection, and the purity of enriched NK was verified via FACS using anti-NK1.1-FITC antibody and anti-CD3-APC antibody. (TIFF) [file ppat.1013315.s002.tiff]

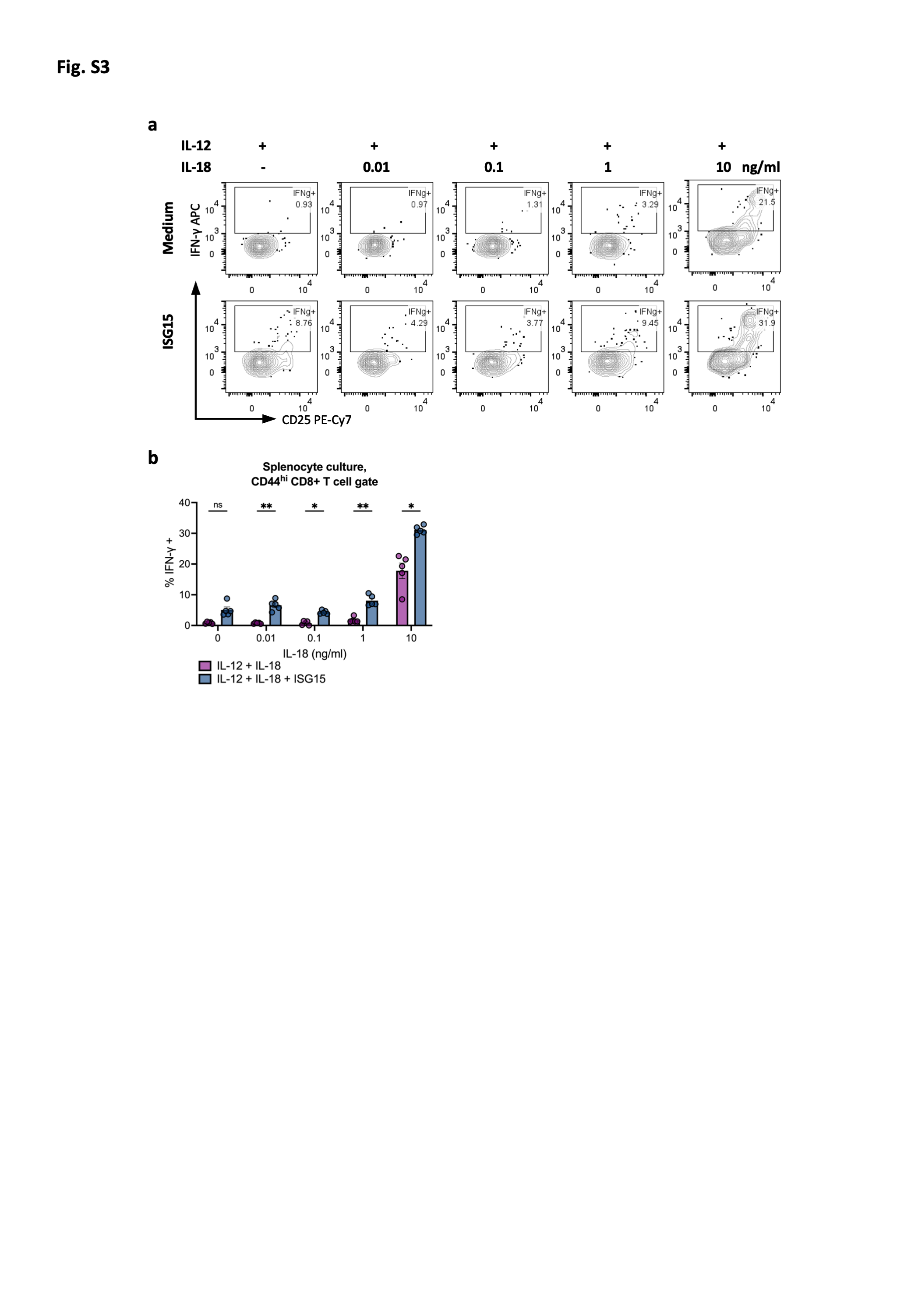

Supplement: S3 Fig — For experimental and statistical details see Fig 2c and 2d. Shown here are whole splenocyte cultures, gated on memory-phenotype CD8+ T cells gated as CD3+ CD8a+ CD44hi. (TIFF) [file ppat.1013315.s003.tiff]

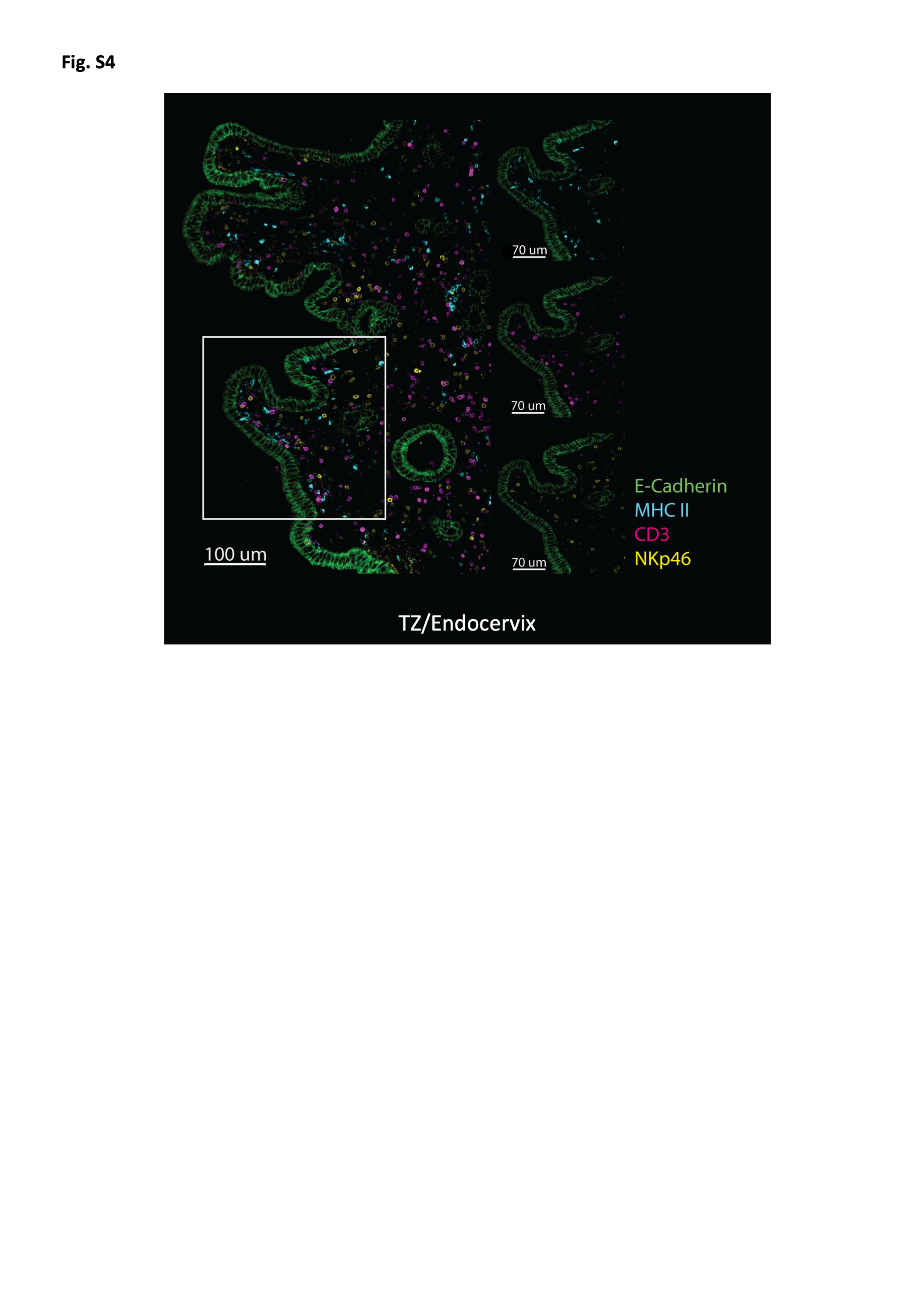

Supplement: S4 Fig — Wild-type mice, either infected with Ct or mock-infected with PBS, were sacrificed at 12 hpi. The entire genital tracts were then isolated and processed into cryosections for immunostaining. The presence of neutrophils (Ly6G), T cells (CD3) and NK cells (NKp46) was analyzed alongside MHC-II expression and the structural markers CD31 and E-cadherin. (TIFF) [file ppat.1013315.s004.tiff]

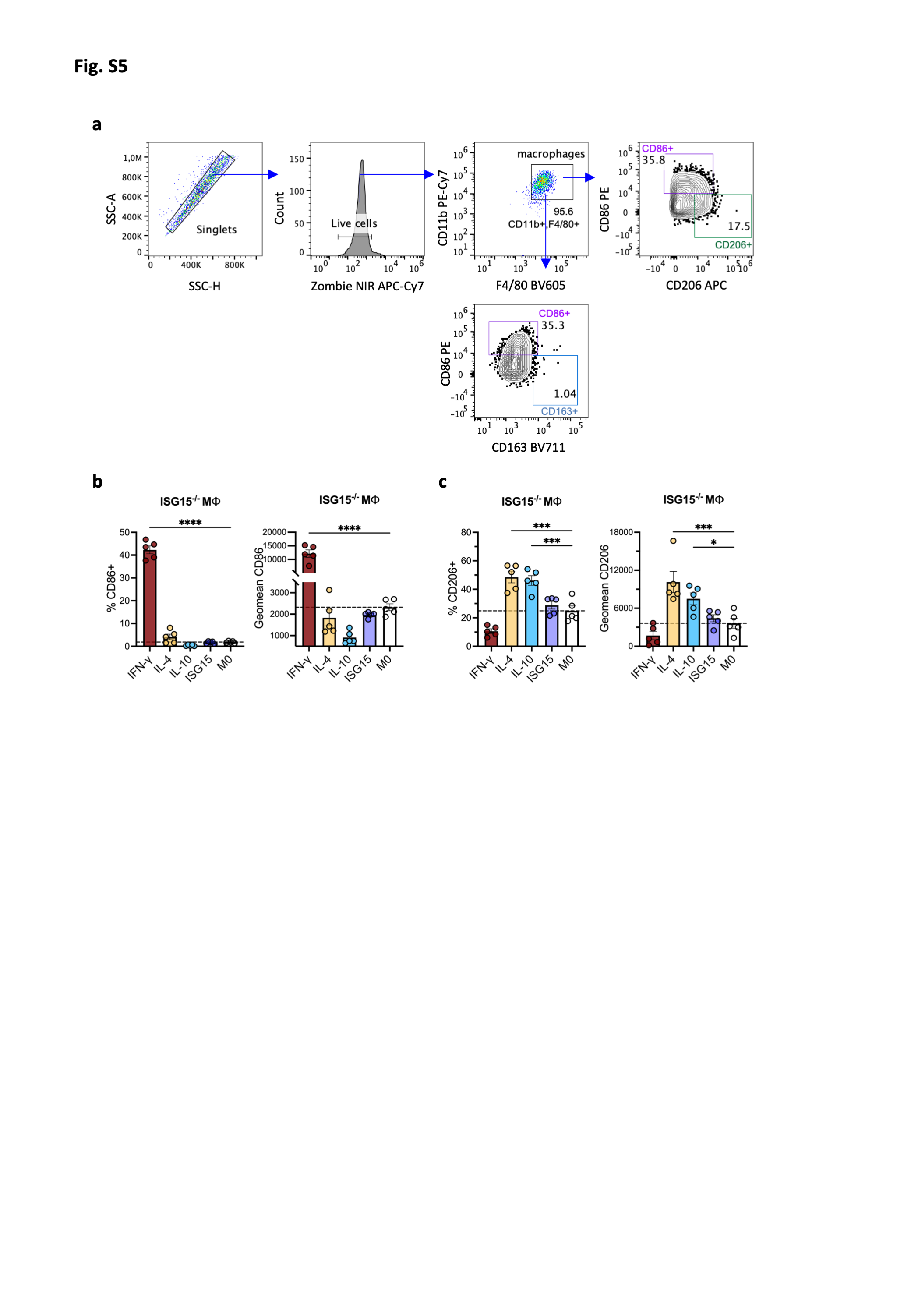

Supplement: S5 Fig — b. The frequency of CD86+ cells and the MFI of CD86 in gated ISG15−/− Mϕ. ISG15−/− Mϕ stimulated as described in Fig 6b, MFI is presented as Geomean values, data were analyzed using one-way ANOVA, n = 5. c. The frequency of CD206+ cells and the MFI of CD206 in gated ISG15−/− Mϕ stimulated as described in Fig 6b. Data were analyzed using one-way ANOVA, n = 5. (Statistical significance is indicated as follows: *p ≤ 0.05; ***p ≤ 0.001; ****p ≤ 0.0001; ns, not statistically significant; n indicates independent biological replicates). (TIFF) [file ppat.1013315.s005.tiff]
