# Supplementary material for: Secreted ISG15 induced by Chlamydia trachomatis infection exerts immunomodulatory effects on IFN-γ defense and inflammation
Source: PLoS Pathog. 2025 Jul 8;21(7):e1013315. doi: 10.1371/journal.ppat.1013315 (PMC12373280; doi:10.1371/journal.ppat.1013315)
Supplement: Attachment — Submitted filename: Response to Reviewers.docx [file ppat.1013315.s008.docx]

**Additional Editor Comments:**

1) Please ensure that the CRediT author contributions listed for every co-author are completed accurately and in full.

At this stage, the following Authors/Authors require contributions: Yongxia Guo, Sigrun V. Stulz, David Kessie, Tommaso Torcellan, Nadine Vollmuth, Qi Tian, Klaus-Peter Knobeloch, Georg Gasteiger, and Thomas Rudel. Please ensure that the full contributions of each author are acknowledged in the "Add/Edit/Remove Authors" section of our submission form.

The list of CRediT author contributions may be found here: <https://journals.plos.org/plospathogens/s/authorship#loc-author-contributions>

Author contributions

Conceptualization: Yongxia Guo.

Formal analysis: Yongxia Guo.

Funding acquisition: Georg Gasteiger, Thomas Rudel.

Investigation: Yongxia Guo, Sigrun Stulz, David Kessie, Nadine Vollmuth, Tommaso Torcellan, Klaus-Peter Knobeloch

Methodology: Yongxia Guo, Sigrun Stulz

Resources: Georg Gasteiger, Thomas Rudel.

Supervision: Georg Gasteiger, Thomas Rudel.

Visualization: Yongxia Guo.

Writing – original draft: Yongxia Guo.

Writing – review & editing: Yongxia Guo, Sigrun Stulz, Georg Gasteiger, Thomas Rudel.

2) Please provide an Author Summary. This should appear in your manuscript between the Abstract (if applicable) and the Introduction, and should be 150-200 words long. The aim should be to make your findings accessible to a wide audience that includes both scientists and non-scientists. Sample summaries can be found on our website under Submission Guidelines:

<https://journals.plos.org/plospathogens/s/submission-guidelines#loc-parts-of-a-submission>

We included an Author summary in the revised manuscript.

3) We noticed that you used the phrase 'data not shown' in the manuscript. We do not allow these references, as the PLOS data access policy requires that all data be either published with the manuscript or made available in a publicly accessible database. Please amend the supplementary material to include the referenced data or remove the references.

We have removed this reference.

4) We do not publish any copyright or trademark symbols that usually accompany proprietary names, eg ©, ®, or ^TM^ (e.g. next to drug or reagent names). Therefore please remove all instances of trademark/copyright symbols throughout the text, including:

- ® on pages: 22 and 23.

- TM on pages: 32 and 47.

We have removed all these copyright or trademark symbols ©, ®, or ^TM^ throughout the text.

5) Please upload all main figures as separate Figure files in .tif or .eps format. For more information about how to convert and format your figure files please see our guidelines:

<https://journals.plos.org/plospathogens/s/figures>

6) Some material included in your submission may be copyrighted. According to PLOSu2019s copyright policy, authors who use figures or other material (e.g., graphics, clipart, maps) from another author or copyright holder must demonstrate or obtain permission to publish this material under the Creative Commons Attribution 4.0 International (CC BY 4.0) License used by PLOS journals. Please closely review the details of PLOSu2019s copyright requirements here: PLOS Licenses and Copyright. If you need to request permissions from a copyright holder, you may use PLOS's Copyright Content Permission form.

Please respond directly to this email and provide any known details concerning your material's license terms and permissions required for reuse, even if you have not yet obtained copyright permissions or are unsure of your material's copyright compatibility. Once you have responded and addressed all other outstanding technical requirements, you may resubmit your manuscript within Editorial Manager.

Potential Copyright Issues:

- Figures 1, 4, 5, and 7. Please confirm whether you drew the images / clip-art within the figure panels by hand. If you did not draw the images, please provide a link to the source of the images or icons and their license / terms of use; or written permission from the copyright holder to publish the images or icons under our CC BY 4.0 license. Alternatively, you may replace the images with open source alternatives. See these open source resources you may use to replace images / clip-art:

- https://commons.wikimedia.org

- https://openclipart.org/.

We created the illustrations in Figures 1, 3, 4, 5, and 7 using BioRender.com, and each figure includes a corresponding license statement in the legend. All images were generated using BioRender’s built-in content under an academic license, which permits use in scientific publications.

7) Please ensure that the funders and grant numbers match between the Financial Disclosure field and the Funding Information tab in your submission form. Note that the funders must be provided in the same order in both places as well. State what role the funders took in the study. If the funders had no role in your study, please state: "The funders had no role in study design, data collection and analysis, decision to publish, or preparation of the manuscript.".

We have included the statement "The funders had no role in study design, data collection and analysis, decision to publish, or preparation of the manuscript.".

**Reviewers' Comments:**

**Part I - Summary**

**Reviewer #1:** The authors performed in vitro and in vivo analyses to examine the role of ISG15 during Chlamydia infection. These studies revealed that murine Ct-infection induced ISG15 production from primary epithelial cells that enhanced NK cell interferon-gamma expression. This inteferon-gamma signaling effect required IL-18 activation of NK cells. The lack of ISG15 in Ct-infected mice modestly reduced bacterial burden and significantly enhanced uterine horn pathology, which was associated with lower interferon-gamma and IL-10 production, respectively. Overall, this is a novel study with well designed experiments and appropriate statistical analyses. The conclusions are well-supported by the data and appropriate citations are used. Overall, the manuscript is of very good merit. To be acceptable for publication, a few minor corrections should be addressed and a couple of additional experiments should be performed that would substantially increase the quality of the manuscript and make for a more comprehensive study.

**Reviewer #2:** In this study, the authors find that Chlamydia trachomatis (CT) infection robustly induces the expression and extracellular secretion of ISG15 locally in vitro and in vivo. They report that ISG15 acts as a critical immunomodulatory cytokine during CT infection. Specifically, they found IL-18, in addition to IL-12, as a novel co-stimulatory cytokine, that acts in synergy with ISG15 to strongly enhance IFNγ production at early stages of. Whereas at later stages, ISG15 promotes IL-10 production to prevent excess inflammatory response and tissue damage. Overall, the experiments are well designed and convincing. However, some of the data seem to contradict the model and correlations are too easily interpreted as causations. While this can be experimentally approached, the authors may alternatively consider to tone down some of their conclusions and adjust the working model.

**Reviewer #3:** This study provides compelling evidence showing the induction of ISG15 in the FGT during Chlamydia infection and the immunomodulatory role of ISG15 in innate immune responses in vitro. Data suggest that secreted ISG15 in the FGT increases IFNg release from NK cells in a IL18-dependent manner. Transcervical Ct infection in ISG15-deficient mice resulted in reduced IFNg at early stage of infection (3 dpi), and increased pathology and bacterial burden at later stage (14 dpi) which correlate with decreased IL-10. This study provides new insights into the critical dual function of ISG15 in modulating host inflammatory responses during Chlamydia infection.

**Part II – Major Issues: Key Experiments Required for Acceptance**

Please use this section to detail the key new experiments or modifications of existing experiments that should be absolutely required to validate study conclusions.

**Reviewer #1:**

(1) Was C.muridarum used in any parallel experiments specifically in mouse cells to confirm ISG15 production in a strain-independent manner using the host species? This would further enhance the quality of the manuscript since it would have further implications for testing in mouse models. Additional in vitro experiments, as performed in Fig 1D-E, are warranted to demonstrate and compare ISG15 production after Cm and Ct infection, at minimum.

Response:

We thank the reviewer for this insightful suggestion. To determine whether ISG15 induction is strain-independent, we conducted additional *in vitro* experiments using mouse female genital tract (mFGT) organoids infected with Cm. Western blot and ELISA analyses showed robust ISG15 expression and secretion, comparable to that observed with Ct infection. These results confirm that ISG15 induction occurs in response to both Cm and Ct in a host-relevant model. These data have been added to the revised manuscript (Fig. 1f; Fig. S1b).

(2) To validate in vitro findings from Figure 6, an ex vivo flow cytometric analysis of CD86/CD163/CD206 expression on macrophages in WT and ISG15-/- mice after infection (e.g. day 3 and 14) is warranted. A co-analysis of marker expression on monocytes (and PMNs, if possible) would further improve the study.

Response:

We thank the reviewer for this thoughtful suggestion. While we agree that ex vivo flow cytometric analysis would provide valuable additional insights, we were unable to perform these experiments due to limitations in animal availability and resources. Given the dynamic nature of macrophage polarization throughout infection, a comprehensive in vivo characterization would require a broader and more longitudinal approach. We plan to pursue this in future studies using techniques such as single-cell RNA sequencing and FACS, which will allow a more comprehensive understanding of ISG15 function in vivo. In the current manuscript, our conclusions in Figure 6 are focused on in vitro findings - including robust phenotypic and functional analyses - which we believe provide strong support for the immunomodulatory role of ISG15 in macrophages and lay a mechanistically relevant foundation for future in vivo work. We have acknowledged this limitation and the need for future in vivo validation in the revised Discussion in line 444-446.

**Reviewer #2:**

(1) The paper shows that significant differences in IFNγ production can be detected between WT and ISG15 KO mFGT at 3dpi (Figure 4d). However, no difference in bacterial burden is observed until 14dpi (Figure 4c) when IFNγ levels are no longer different between ISG15 WT and KO mFGT (Figure 4d). These data seem to contradict the proposed model that diminished IFNγ production in ISG15 KO mice is responsible for the elevated CT burden in ISG15 KO mice. To prove causality IFNγ blocking antibodies could be used. The expected outcome would be that the difference in CT burden between WT and ISG15 KO mice would disappear once IFNγ signaling is blocked. Alternatively, the authors should acknowledge that the data actually contradicts their model and come up with a new model to explain the ISG15 KO burden phenotype.

Response:

We thank the reviewer for the insightful comment. IFNγ limits Chlamydia replication and the production of infectious progeny, with effects that may accumulate over multiple infection cycles. Therefore, the reduced IFNγ levels observed at 3 dpi in ISG15⁻/⁻ mice may not immediately impact bacterial burden but likely contribute to the higher load seen at 14 dpi. To bridge this temporal gap, we assessed both IFNγ levels and bacterial burden at 7 dpi. At this time point, ISG15^⁻/⁻^ mice exhibited significantly reduced IFNγ and already showed an increase in bacterial burden, supporting our model. These new results have been incorporated into the revised Fig. 4c and 4d.

While IFNγ is not the only factor involved in bacterial clearance, our data support a role for ISG15 in promoting early IFNγ responses that shape the course of infection. We now acknowledge the correlative nature of these findings in the revised Discussion in line 420-422.

(2) The authors propose that ISG15 signals to nearby NK cells in mFGT to enhance the IFNγ response during CT infection. In support of this model the paper provides an image that possibly shows NK cells near epithelial cells in WT mFGT at 12hpi versus mock-infected mFGT (Figure S4). However, it is not clear why 12hpi was chosen as a timepoint and whether NK cells were also present in ISG15 KO mFGT. The authors should consider not showing Figure S4, as these data lack controls and are not quantified. Maybe a better approach would be to perform FACS analyses on immune cells present at WT versus ISG15 KO mFGT during C.trachomatis infection to strengthen their findings of NK cell recruitment/infiltrate to infected the epithelium

Response:

We selected 12 hpi based on previous studies (Tseng C.T., Ref. 28), which reported early NK cell activity in the genital tract following *C. muridarum* infection. This time point was chosen to visualize initial immune-epithelial interactions. To strengthen our findings, we performed FACS analysis to quantify NK cell infiltration (live CD45⁺CD3⁻CD11b⁺NK1.1⁺CD49b⁺) in WT and ISG15⁻/⁻ mice at 24 hpi. Infection significantly increased NK cell infiltration, but no significant difference was observed between WT and ISG15⁻/⁻ group. These data indicate that ISG15 does not affect NK cell recruitment, while supporting quick response and involvement of NK cells in the genital tract during Chlamydia infection. These data are now included in revised Fig. 4g–h.

(3) The authors should include all necessary controls (untreated/uninfected control, IFNγ alone, ISG15 alone, and C. trachomatis infection alone) when assessing cytokine secretions (Figure 6 f-h).

Response:

We thank the reviewer for this suggestion. In this set of experiments (Figure 6f–h), we aim to assess how IFN-γ modulates macrophage responses to Ct infection, and how ISG15 influences this effect. Since macrophages *in vivo* are rarely exposed to infection without concurrent cytokine signals, we focused on infection-driven responses under physiologically relevant conditions. While additional controls such as uninfected/untreated, IFN-γ alone, or ISG15 alone could offer further mechanistic detail, we believe our current experimental design sufficiently demonstrates that ISG15 restrains the IFN-γ–driven pro-inflammatory response in macrophages.

**Reviewer #3:**

(1) The authors show in Fig. 2e that ISG15 regulates NK cell IFNg production in a cell-extrinsic manner. However, in the organoids culture system in Fig.3b, it appears that ISG15 secreted from the organoids directly stimulates NK cells, as no other cell type exist in this system. Please explain the discrepancy.

Response:

We thank the reviewer for this insightful comment. In the organoid - NK cell co-culture system (Fig. 3b), the primary components are epithelial cells and >85% enriched NK cells (see Methods and Fig. S2), with minimal residual immune cells that are unlikely to account for the observed IFN-γ production. Notably, epithelial cells can produce IL-18 in response to Chlamydia infection, as demonstrated in multiple epithelial models and Chlamydia serovars (Ref. 51), and IL-18 is increasingly recognized as an epithelium-derived cytokine contributing to mucosal immunity (Ref. 30).

To investigate this experimentally, we measured IL-18 levels in the supernatants of infected mFGT organoids and observed elevated secretion in both WT and ISG15⁻/⁻ organoids (revised Fig. 3c), suggesting that NK cell activation in this system is driven by both ISG15 and epithelial-derived IL-18. We have incorporated the supporting data and references in the revised manuscript (Fig. 3c, Ref. 51).

(2) The macrophage data in Fig. 5 and 6 relies heavily on CD163 as a M2 marker. However, data in Fig. 5b does not support CD163 as a good murine M2 marker (as opposed to human macrophage). The positive control IL-4 does not induce a prominent CD163+ population. In contrast, CD206 works better as a M2 marker, but is not pursued. There is also a lack of functional readouts such as iNOS and Arg in support of M1/M2 phenotype.

Response:

We thank the reviewer for this important comment. As noted, CD163 is not a well-established M2 marker in murine macrophages and is more commonly used in human studies. Consistent with this, IL-4 did not robustly induce CD163 expression in our system (Fig. 5b). Given the heterogeneity and phenotypic diversity of M2 macrophages (Ref. 64), macrophage polarization requires the integration of multiple surface markers and functional outputs, rather than relying on a single marker. To address this, we used a marker panel--CD86, CD206, and CD163--to capture broader phenotypic patterns, rather than relying on a single CD163 (Fig. 5c–e):

M1 (IFNγ-induced): CD86^^hi^, CD206^^lo^, CD163^^neg^

M2 (IL-4-induced): CD86^^lo^, CD206^^hi^, CD163^^lo^

M2 (IL-10-induced): CD86^^neg^, CD206^^hi^, CD163^^lo^

ISG15-induced: CD86^^lo^, CD206^^int^, CD163^^hi^

ISG15-treated macrophages exhibited a distinct profile that partially overlaps with M2-like features. Functional assays (Fig. 6) showed that ISG15 reduced pro-inflammatory cytokines (IL-6, TNF-α) and increased IL-10, supporting an anti-inflammatory, M2-like phenotype. Although iNOS and Arg1 were not assessed, we believe the combined phenotypic and functional data provide strong evidence that ISG15 promotes M2-like polarization. These points have been clarified in the revised manuscript, along with the inclusion of a relevant reference (Ref. 64).

(3) In vivo phenotyping of IFNg+ NK cell and M1/M2 macrophages in WT and ISG15-/- mice would significantly strengthen the study.

Response:

We thank the reviewer for this valuable suggestion. We added two experiments: (1) quantification of IL-18 in infected mFGT organoids (see Reviewer #2, Comment 2), and (2) flow cytometric analysis of NK cell infiltration at 24 hpi. These results support our model that epithelial-derived ISG15 and IL-18 synergize to activate NK cells and promote IFNγ production (revised Fig. 3c, Fig. 4g-h).

While in vivo profiling of M1/M2 macrophages would provide additional insight, we were unable to perform these experiments due to resource limitations and the substantial *in vivo* work already included (e.g., bacterial burden, IFNγ levels at 7 dpi, and NK cell infiltration). Nonetheless, our in vitro data (Figs. 5–6) strongly support ISG15’s role in promoting an anti-inflammatory, M2-like macrophage phenotype. We have noted in vivo validation as an important future direction in the revised Discussion in line 444-448.

**Part III – Minor Issues: Editorial and Data Presentation Modifications**

Please use this section for editorial suggestions as well as relatively minor modifications of existing data that would enhance clarity.

**Reviewer #1:**

Abstract - “ISG15 can exist as a covalently conjugated protein or as an unconjugated cytokine.” Please revise to something like ‘can exist in unconjugated form or conjugated via ISGylation to other host proteins’ to better detail its function.

Response: We have revised this sentence.

Line 72 – change ‘aiming’ to ‘aimed’.

Response: We have revised this.

Line 87-89 - The writing could be better parsed regarding the findings from citation 26 to more clearly state interferon-gamma redundancy occurs specifically in the context of dissemination.

Response: We have re-parse this sentence.

Line 234 – It states that hydrosalpinx was evaluated. Ct causes minimal oviduct disease in mice and is usually analyzed significantly past day 14 post-infection. Uterine horn pathology was assessed but oviduct scores are absent. If no hydrosalpinx/oviduct dilation was observed, then this should be stated.

Response: We now state ‘although neither hydrosalpinx nor oviduct dilation was observed’.

Line 286 – Please rephrase to indicate that citations 58,59 are from other disease models. The writing could be misconstrued that it is from a Chlamydia model.

Response: We have revised this sentence.

Line 307 – Like above, please specify if observations are made in Chlamydia or other pathogen models. Please review the entire manuscript to better clarify these instances.

Response: We have revised this sentence.

Figure 6 - The IFNg label on the x-axis is cut off in panels b-d.

Response: We have corrected the IFN-γ label on the x-axis.

Lines 664-669. The methods suggest macrophages were analyzed ex vivo after murine infection. If so, this would help address the above major critique regarding analyzing macrophage marker expression in mice post-infection.

Response: We have removed the corresponding text from the Methods section for consistency, as ex vivo macrophage analysis was not included in the final study.

**Reviewer #2:**

1. One key notion the authors were trying to demonstrate was the low level of IL-18 in ISG15 stimulation of IFNγ (Figure 2d-e). It would provide more solid ground for this hypothesis if IL-18 secretions during infections were assessed.

Response: We thank the reviewer for this comment. To directly address this point, we measured IL-18 levels in supernatants from infected mFGT organoids. IL-18 was significantly elevated in both WT and ISG15⁻/⁻ groups compared to uninfected controls (revised Fig. 3c). These results support our model that epithelial-derived IL-18 contributes to ISG15-mediated IFNγ induction in NK cells. We have included these supporting data and references in the revised manuscript (Fig. 3c, Ref. 51).

2. The authors showed that intracellular ISG15 is dispensable for the C.trachomatis infection, and the conjugation of ISG15 is not the focus of the study. However, showing and assessing the free ISG15 levels in all SDS-PAGE analyses alone are not sufficient to rule out changes and involvements of ISG15-conjugation. The authors should consider providing full blots containing the conjugated upper bands in the supplemental figure to further support their findings.

Response: We have now included the full blots showing ISG15 and its conjugated forms to address this point. No obvious ISG15-conjugated upper bands were observed, supporting our conclusion that ISGylation is not a major feature under our experimental conditions. The full blots of ISG15 have been added to the revised Figure 1.

3. The authors mentioned that basal level of gamma between WT and ISG15 KO (line 246-247) without citing any previous study. Was this data not shown or previously established and should be cited?

Response: The basal level of IFN-γ mentioned in this sentence is supported by our data presented in Fig. 4d, which is now cited in the text for clarity.

4. The authors proposed that at later time of the infection, ISG15 acts through IL-10 to modulate macrophage functions for tissue repair. However, the authors only assessed IL-10 and IFNγ levels (Figure 4d, 4g-i) and not other immunomodulatory cytokines. Supplementing IL-10 exogenously and monitor the effect on animal pathology in ISG15 KO vs WT mice would provide evidence for a causal link. Without such data, the authors should acknowledge the limitations of their correlative studies.

Response: We thank the reviewer for this comment. While our findings suggest a correlation between ISG15 and IL-10 in limiting immunopathology, we acknowledge that this relationship has not been directly tested. Our observations are consistent with the previous study (Ref. 48) describing an anti-inflammatory ISG15/IL-10 axis in other disease contexts. We have now noted this limitation in the revised Discussion (lines 446–448), highlighting that the in vivo interaction between ISG15 and IL-10 remains to be fully elucidated.

5. Lastly, the authors demonstrated that ISG15 stimulation of macrophages in vitro leads to the polarization of M2-like macrophages (7 days of differentiation followed by 24h cytokine stimulation). They propose these M2-like macrophages (CD163hi) account for the tissue damage control at late infection. The authors may consider using FACS analyses to demonstrate macrophages present in the WT but not ISG15 KO mFGT to have similar signatures particularly at 7, 14, and 28 dpi.

Response: We thank the reviewer for this comment. As noted in our response to Reviewer #3 (Part II), we agree that in vivo profiling of M1/M2 macrophage would provide additional insight. However, due to resource limitations and the extensive in vivo work already completed - we were unable to incorporate macrophage phenotyping in the current study. We consider in vivo validation of this observation an important direction for future work.

**Reviewer #3:** (No Response)
